# Research on the Polishing Process of Wolter-I Type Grazing Incidence Mirrors

**DOI:** 10.1371/journal.pone.0317239

**Published:** 2025-03-13

**Authors:** Song Ding, Shuhai Yao, Liwei Sun, Fangpu Feng, Dongling Chen

**Affiliations:** 1 Faculty of Engineering, Changchun Normal University, Changchun Jilin, People’s Republic of China; 2 Jilin Diesel Engine Co., Ltd, Changchun Jilin, People’s Republic of China; Manipal Academy of Higher Education, INDIA

## Abstract

As the demand for solar X-ray observation devices continues to rise, the Wolter-I type grazing incidence mirror has emerged as a critical component in these instruments, particularly for high-precision imaging. This mirror efficiently focuses X-rays, enabling astronomers to detect fainter celestial signals. It plays a key role in providing essential data for understanding the origin and evolution of the universe. The operating principle of the Wolter-I type grazing incidence mirror is based on grazing incidence reflection. This reflection guides X-rays to a focal point through a specific surface structure, enabling high-resolution imaging. This paper presents the design of a super-precision optical processing system for the Wolter-I type mirror. The system features a coaxial Confocal structure, consisting of a rotating parabolic surface and a rotating hyperbolic surface. It also includes a radial adjustment fixture and an automatic polishing fluid supply device. The paper outlines the imaging principles of the Wolter-I mirror, analyzes the impact of surface shape accuracy on imaging performance, and selects microcrystalline glass as the mirror substrate. Using the custom-designed processing system, the rough-turned workpiece undergoes several steps. After diamond wheel grinding, the workpiece is subjected to 160 hours of rough polishing. It then undergoes 720 hours of fine and super-fine polishing, using cerium oxide polishing fluids with particle sizes of W2, W1, and W0.8, respectively. The final surface shape accuracy of the mirror is characterized by a peak-to-valley (PV) value of 253 nm, a root mean square (RMS) value of 3.5 nm, and a root mean square roughness (Rq) of 4.6 nm. These values meet the requirements for composite extreme ultraviolet-soft X-ray telescopes. Experimental results show that the designed super-precision optical processing system effectively improves surface shape accuracy. It is well-suited for processing the unique internal surfaces of Wolter-I type grazing incidence mirrors. This system enhances the mirror's imaging performance and lays a solid foundation for future high-resolution X-ray astronomical observations. Future research will focus on optimizing the processing techniques further, exploring the impact of different materials on imaging quality, and developing more advanced optical systems to meet emerging observational needs.

## 1. Introduction

The Wolter-I type grazing incidence mirror plays a critical role in space X-ray observation equipment. The renowned “Chandra” satellite[1], developed by the United States, utilizes Wolter-I type grazing incidence mirrors to focus high-energy X-rays, achieving high-resolution observations of phenomena such as black holes, galaxy clusters, and supernova remnants. The XMM-Newton satellite[2], launched by the European Space Agency, also employs a Wolter-I mirror system. This system includes three independent telescope units capable of conducting multi-band X-ray observations, with tasks that encompass the study of black holes and pulsars. Japan’s Hitomi satellite[3], designed based on the Wolter-I concept, despite losing contact post-launch, retains significant scientific value due to its mirror design. China's Insight satellite is the first hard X-ray modulation telescope [4], although it primarily employs modulation technology, some modules utilize grazing incidence techniques. Furthermore, several developing X-ray observation satellite projects in China are expected to adopt Wolter-I type mirrors more extensively. Therefore, researching the manufacturing processes of Wolter-I type grazing incidence mirrors is of paramount importance. The primary manufacturing processes include mandrel replication and polishing techniques. However, the surface shape accuracy of Wolter-I mirrors produced through mandrel replication typically does not match that achieved through polishing. This discrepancy arises from factors such as material shrinkage and deformation, stress-induced warping, insufficient surface smoothness of the mandrel, lack of post-processing, and limitations in manufacturing precision [5]. Consequently, improving the polishing process for Wolter-I type mirrors has become a focal point of research.

The pursuit of enhancing the surface shape accuracy of Wolter-I type grazing incidence mirrors has been a long-standing objective, as it not only impacts the precision of astronomical observations but also directly influences the depth and breadth of scientific research [6–9]. In 1952, German scientist Wolter proposed the design principle for a grazing incidence X-ray imaging system based on secondary rotationally symmetric surfaces [10]. However, due to the limitations of manufacturing techniques and technology at the time, such grazing incidence imaging systems did not see widespread application in X-ray astronomy until the 1970s. Bernd Aschenbach et al. developed a grazing incidence telescope for the X-ray astronomy satellite ROSAT, which includes both a verification model and a flight model. This telescope consists of a quadruple nested Wolter I mirror assembly, with an aperture of 84 cm and a focal length of 240 cm [11]. Philippe et al. developed an off-axis Wolter lens with a surface roughness of 0.3 nm RMS [12]. H. K. H. Hwang et al. produced an off-axis Wolter lens with a focal length of 3500 mm, achieving a resolution of 4 microns [13]. R. D. Ge et al. developed a novel off-axis Wolter-I lens with a focal length of 4800 mm and a resolution of 6 microns, utilizing advanced nano-manufacturing technologies to improve surface shape accuracy [14]. K. Y. Lee et al. created a Wolter-I lens using laser machining technology, achieving a focal length of 4000 mm and an imaging resolution of 5.5 microns, with enhanced reflectivity through optimized coating techniques [15]. M. R. R. S. Okamoto et al. researched multi-layer coated Wolter-I lenses, which improved the effective collection of X-rays, also with a focal length of 4000 mm [16]. Z. L. Wang et al. developed high-performance Wolter-I lenses using multi-layer optical coating technology, achieving a focal length of 4500 mm and an imaging resolution of 4.5 microns, significantly enhancing the effective collection capability of the mirrors [17]. The SPring-8 facility reported a single-piece Wolter lens provided by Nakanishi Optical Co., featuring a grazing incidence angle of 1.2°, an object distance of 250 mm, and a magnification of 38 × , achieving a final surface shape precision of 3 nm RMS, with imaging spot sizes of 0.32 microns (horizontal) and 3.4 microns (vertical) [18]. S. T. Y. Tsai et al. produced a composite Wolter lens that combined different materials to optimize imaging performance, achieving a resolution of 5 microns [19]. Yuzo Mori et al. developed a novel CNC plasma chemical vaporization (CVM) machine that efficiently manufactures ultra-high precision X-ray mirrors without producing deformation layers, achieving a flat mirror with a PV value of 22.5 nm [20]. James H. Underwood et al. employed sputtering technology to create a multi-layer interference mirror for reflecting carbon K X-rays (wavelength λ =  4.48 nm), composed of 76 layers of tungsten, each 0.765 nm thick, interleaved with 1.510 nm thick carbon layers, on a silicon substrate [21]. By curving the substrate, this LSM formed a concave mirror with a radius of approximately 1.1 meters, utilized in optical setups for imaging grids illuminated by X-ray sources. Tianyi Wang et al. proposed a novel robust iterative surface extension (RISE) method that effectively addresses multiple issues present in traditional methods through data fitting strategies [22]. RISE models graphical errors in the CA using orthogonal polynomials, ensuring that only correctable errors are fitted and extended. Additionally, by integrating boundary conditions, an iterative improved residence time is proposed to compensate for errors arising during the extension and deconvolution processes. Yuichiro Ezoe [23] et al. developed a novel ultra-lightweight high-resolution X-ray micropore optical component, initially fabricated using deep reactive ion etching (DRIE) or X-ray LIGA processes to create curved micropore structures, followed by smoothing treatments on the sidewalls of the microstructures using magnetic field-assisted polishing and hydrogen annealing techniques to enhance the reflectivity of the mirrors. To focus parallel X-ray beams from celestial sources, these structures are elastically or plastically bent into spherical shapes. Finally, the curved structures are stacked to form multi-stage X-ray telescopes. The study reported results from manufacturing silicon and nickel X-ray mirrors using DRIE and LIGA processes, confirming the X-ray reflectivity of both mirror types for the first time, with measured RMS roughness values of 5 nm for the silicon mirror and 3 nm for the nickel mirror, indicating good surface quality. D. Laundy [24] et al. proposed a novel beam shape variation concept aimed at rapidly adjusting the size and shape of focused X-ray beams. Their method involves segmenting the surface of an elliptical parabolic mirror into multiple laterally independent channels, each endowed with additional longitudinal height profiles to shape the X-ray beam into a top-hat profile. This design allows for variable beam shapes within the focal plane.

Most of the aforementioned literature focuses on improving processes rather than enhancing polishing equipment. Upgrading polishing machines is a crucial step in enhancing the manufacturing precision and optical performance of Wolter-I type grazing incidence mirrors. This not only addresses existing deficiencies in current research but also promotes further advancements in fields such as X-ray astronomy. This paper presents improvements made to existing polishing machine equipment, including the independent design of an automatic supply device and a workpiece clamping device. The study conducts a theoretical analysis of the polishing process for Wolter-I type grazing incidence mirrors, determining the algorithms for residence time calculations and planning polishing trajectories. It then performs CCOS polishing experiments on the Wolter-I type mirrors and compares the surface shape accuracy of the mirrors processed with the upgraded equipment to those produced with the previous polishing devices, demonstrating a significant enhancement in the surface shape accuracy of the Wolter-I type mirrors following the improvements.

## 2. X-ray grazing incidence imaging

### 2.1. X-ray grazing incidence critical angle

X-rays have a very short wavelength and strong penetrating ability compared to conventional visible light. Ordinary prisms, lenses, and plane mirrors cannot effectively reflect or focus X-rays, as their refractive index is close to (or slightly less than) 1. When X-rays incident on the surface of a medium at a small angle, total reflection can occur. To focus or image X-rays, the incident angle must be very small. This angle decreases with increasing X-ray energy, while reflectivity decreases as the angle of incidence increases. The grazing angle reduces as X-ray energy increases, and the reflectivity decreases with an increase in the grazing angle.When X-rays strike the mirror surface with an angle of incidence smaller than a specific critical angle, total reflection occurs. According to Snell’s law, the critical angle ($\theta_{c}$ ) can be determined. The change in atomic density ($n_{a}$) is slow and nearly constant. The atomic number ($f_{1}$) is approximately equal to the material's atomic number *Z.* The critical angle ($\theta_{c}$) of a medium primarily depends on the wavelength *λ* and the atomic number *Z*.


$$\theta_{c}=\sqrt{\frac{n_{a}r_{e}\lambda^{2}f_{1}}{\pi }}$$ (1)

$n_{a}$: Atomic density in materials

$r_{e}$: Classical radius of the electron

*λ*: X-ray wavelength

$f_{1}$: Complex scattering factors for atoms

### 2.2. Wolter-I grazing incidence optical system

The Wolter-I type swept-incidence optical system consists of parabolic and hyperbolic surfaces. X-ray light, incident at infinity, is reflected first by the parabolic surface and then by the hyperbolic surface. This sequence of reflections focuses the light onto the optical system's focal plane, creating an image.The Wolter-I optical system has minimal impact on light energy concentration. It effectively corrects off-axis aberrations, thereby improving the imaging quality of the system.

Equation for the face shape of an X-ray grazing incidence reflector:


$$z=\frac{cr^{2}}{1+\sqrt{1-\left(k+1\right)c^{2}r^{2}}}$$ (2)

*z*: Surface Vector Height

*c*: Curvature of a paraboloid or hyperboloid vertex

*r*: Radial radius of the coordinate system

k=−1: The surface shape is parabolic

k<−1: the surface shape is hyperbolic

In accordance with the requirements of the composite extreme ultraviolet-soft X-ray telescope and based on the design of the GOES satellite launched by the United States, the Wolter-I type swept-incidence design is applied for the soft X-ray band (0.6 nm-6 nm). The design uses an overall undivided structure. The aspherical equations are given in Eq. 3 and 4.

Parabolic equation:


$$y=\frac{1}{2}Cx^{2}$$ (3)

Where C is the vertex curvature of the parabola, C = 0.411275529.

Hyperbolic equations:


$$\text{y}=\frac{{\text{Cx}}^{2}}{1+\sqrt{1-\left(\text{k}+1\right){\text{x}}^{2}}{\text{C}}^{2}}$$ (4)

Where C is the vertex curvature of the hyperbola, C =  0.4097588, and k is the eccentricity coefficient, k =  –1.007424.

The swept-incidence telescope is a specialized Cassegrain system. According to the technical specifications, it must operate within the wavelength range of 0.6 nm to 6 nm. The aspherical reflector has a length of 102 mm, with a joint spacing of 3 mm. The diameter of the parabolic/hyperbolic intersecting plane is 160 mm.

## 3. Optical grazing incidence mirror surface accuracy

### 3.1. Surface roughness

X-ray wavelengths are in the nanometer range. When X-rays incident on an optical surface, surface errors can scatter or alter the light propagation path. This, in turn, affects the imaging quality of the optical system.

According to Harvey-Shack scalar scattering theory, the bi-directional reflectance distribution function (BRDF) is used to analyze the impact of mirror surface roughness on the imaging quality of a soft X-ray grazing incidence telescope. This is expressed in Equation 5:


$$BRDF=Q\cdot K\left(\gamma_{i}\right)\frac{4\pi^{2}}{\lambda^{4}}{\left(\cos\theta_{i}+\cos\theta_{s}\right)}^{2}\cdot \frac{\sigma_{rel}^{2}}{\sigma_{s}^{2}}PSD\left(f_{x},f_{y}\right)$$ (5)

$K\left(\gamma_{i}\right)$ *: Reformulation factor*

$\theta_{i}$ *: Angle of incidence*

$\theta_{s}$ *: Scattering angle*

$\sigma_{\text{rel}}$ *: Effective root-mean-square surface roughness*

$\sigma_{s}$ *: Intrinsic Root Mean Square Surface Roughness*

$f_{x}$*: Spatial frequency* x component

$f_{y}$*: Spatial frequency* y component

### 3.2. Surface shape error

Face shape errors originate from two main sources: processing and installation, and thermal loads that increase the mirror surface temperature. Aspheric optical mirrors are theoretically manufactured with high precision, achieving micrometer-level accuracy and minimal aberrations. However, at the nanometer scale of X-ray wavelengths, face shape errors become significant. These errors affect focusing in different directions.In the meridian direction, the grazing incidence angle of the incident light on the optical surface is denoted as *θ*. The change in the surface's normal direction is *δ*, which causes a corresponding change of 2δ in the outgoing light direction. This results in a displacement in the optical image.


$$\Delta S_{meridian}\text{=r}\prime 2\delta +o\left(2\right)\approx r\prime 2\delta$$ (6)

Where $\Delta S_{meridian}$ is the surface shape error in the meridian direction, is the change in the direction of the normal of the optical surface, *δ* is the angle at which the direction of the outgoing light changes.

For focusing in the arc-vector direction, the direction normal to the optical plane changes γ, and the direction of the outgoing light changes 2*γ*, but the displacement of the optical image in the arc-vector direction is:


$$\Delta S_{Arcsaddle}\text{=r}\prime \sin2\theta \gamma$$ (7)

Where $\Delta S_{Arcsaddle}$ is the surface shape error in the sagittal direction, is the change in the direction of the normal of the optical surface, *θ* is the grazing incidence angle of the incident light on the optical surface, *γ* is the direction normal to the optical plane.

The grazing incidence angle is small. For the same face shape error, the image dispersion caused by focusing in the arc vector direction is much smaller than that in the meridian direction. Therefore, the primary concern is the image dispersion caused by focusing in the meridian direction. To reduce this dispersion, it is essential to improve face shape accuracy. This can be achieved by selecting materials with low thermal deformation and adjusting processing methods to control face shape errors at the nanometer level.

## 4. Optical swept-incidence mirror processing principle

### 4.1. Automatic supply of polishing liquid device

Glass and other brittle materials have very low toughness, typically in the range of 10 ⁻ ² to 10 ⁻ ³ for metals. These materials are prone to breaking and cracking during mechanical processing. Ordinary cutting or grinding methods cannot achieve a smooth surface on brittle materials. However, the polishing mechanism of optical components shows that the mechanical removal effect during glass polishing is objective and effective.

**Fig 1:**
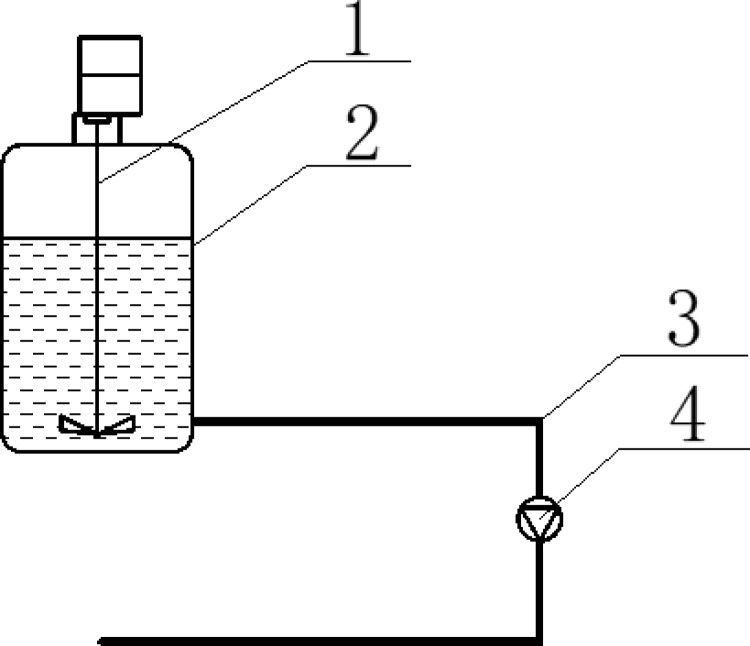
Automatic polishing fluid supply device.

To enhance precision and efficiency in mirror surface processing, an automatic polishing liquid supply device was designed for ultra-smooth finishing. This device, shown in Fig 1, includes four main components: a stirring device, a liquid storage cylinder, a liquid delivery pipe, and a peristaltic pump. The polishing machine is placed on a table with a liquid storage cylinder containing a micro-motor. A stirring blade is mounted on the motor shaft. When powered, the micro-motor rotates, driving the blade to mix the polishing liquid uniformly. A small opening at the bottom of the cylinder connects to a delivery pipe that directs the polishing liquid to the center of the polishing disc. The delivery pipe includes a peristaltic pump to fine-tune the flow rate and timing of the liquid. This setup ensures precise control over the liquid discharge interval and amount, maintaining a uniform layer thickness during the mirror surface polishing process.

1-mixing plant 2-liquid storage barrel

3-infusion tube 4-peristaltic pump

### 4.2. Remove function

Flat, spherical and aspherical surfaces with low steepness can be processed using flat rotation or planetary motion of the grinding head. However, for the Wolter-I design, which has a special near-columnar structure, flat or planetary grinding is no longer suitable. In this case, rotary deflection of the grinding head is used to machine the inner surface of the column.

Under certain assumptions, the relationship between the amount of material removed from mechanically chemically polished optical surfaces and various process parameters can be described by the Preston equation.


$$\frac{dz}{dt}=kvp$$ (8)

Where *p* is the pressure distribution function on the surface, *v* is the velocity distribution function on the surface, $\frac{dz}{dt}$ is the amount of material removed per unit of time at any point in the contact area between the grinding head and the workpiece, p is the pressure distribution function on the surface, *v* is the velocity distribution function on the surface, *K* is a constant of proportionality, which is determined by all factors except speed and pressure, establishing a linear relationship between the amount of material removed, pressure and instantaneous speed.

For ease of discussion, the mirror of the Wolter-I structure is simplified to an ellipsoidal model and the grinding head is simplified to a spherical model. The model is shown in the following equation:

Spherical equations:


$$x^{2}+y^{2}+{\left(z+l\right)}^{2}=r^{2}$$ (9)

Elliptic surface equations:


$$\frac{y^{2}}{a^{2}}+\frac{z^{2}}{b^{2}}=l$$ (10)

Where *r* is the radius of the grinding head, *a* is the distance from the long axis of the ellipsoid surface, *b* is the distance from the short axis of the ellipsoid surface, and l is the distance between the center of the ball and the center of the ellipsoid.

**Fig 2:**
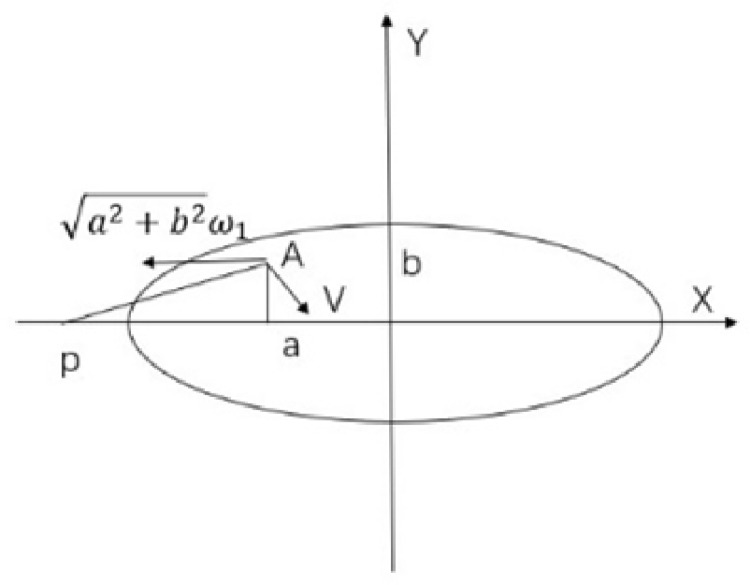
Schematic diagram of velocity synthesis at any point on the contact surface.

If the contact surfaces of the workpiece and the grinding head are spread out in the X-Y coordinate system, the area removed by the grinding head when the workpiece is stationary is an ellipse. When the workpiece is rotating, it can be understood that the grinding head is rotating and moving along the Y-axis at a speed of $\sqrt{a^{2}+b^{2}}\omega_{1}$ The workpiece is rotating, which can be interpreted as the grinding head rotating while moving along the Y-axis with a uniform linear velocity. This is shown in Fig 2.

Since the workpiece is a cylindrical inner surface, it is reasonable to consider the superposition of velocities in the X-Y coordinate system after leveling. The isovelocity line on the contact surface is on the arc centered at point p. For any point A, the velocity distribution of the grinding head rotation superimposed on the workpiece rotation is:


$$v=\sqrt{v_{x}^{2}+v_{y}^{2}}=\sqrt{(a^{2}+b^{2})+h^{2}\omega_{2}^{2}+2\sqrt{a^{2}+b^{2}}\omega_{1}\omega_{2}\sin\beta }$$ (11)

Among them:


$$\tan\beta =\frac{x}{y+\sqrt{a^{2}+b^{2}-x_{0}^{2}}/\tan\varphi }$$ (12)

And because $\sqrt{a^{2}+b^{2}}\omega_{1}$ =  $v_{workpiece}$ =  $\frac{d_{y}}{d_{t}}$. So the depth of removal for one rotation of the workpiece is:


$$\Delta =\text{k}\int pv\cdot \text{dt}=\text{k}\int_{-y_{x}}^{y_{x}}p_{0}\sqrt[{n}]{1-f\left(x,y,z\right)}*\text{dt}=\text{k}\sqrt{1+\frac{h^{2}}{a^{2}+b^{2}}\rho^{2}+\frac{2h}{\sqrt{a^{2}+b^{2}}}\rho \sin\beta }\;dy$$ (13)

Where $\rho =\frac{\omega_{2}}{\omega_{1}}$ is the ratio of the angular velocity of the abrasive head to that of the workpiece, and $y_{x}$ is the half-width of the abrasive head-workpiece contact area along the Y-axis corresponding to different x's, * means multiplication.

From this, it can be determined that the feature removal function at $x_{i}$ of the grinding head is the distribution of the removal depth per unit time with *x*:


$$\Delta R\left(x_{i},x\right)=\Delta \cdot \omega_{1}=\text{k}\omega_{1}\int_{-y_{x}}^{y_{x}}p_{0}\sqrt[{n}]{1-f\left(x,y,z\right)}*\sqrt{1+\frac{h^{2}}{a^{2}+b^{2}}\rho^{2}+\frac{2h}{\sqrt{a^{2}+b^{2}}}\rho \sin\beta }\;dy$$ (14)

Polishing is a long and complex process, during which the Wolter-Ⅰ type swept-incidence reflector should be tested several times, and according to the results of each test, the processing parameters should be adjusted to ensure that the grinding head can accurately remove the protruding points on the mirror surface, reduce the surface roughness of the mirror surface, and make the removal function contour close to the Gaussian distribution function.

**Fig 3:**
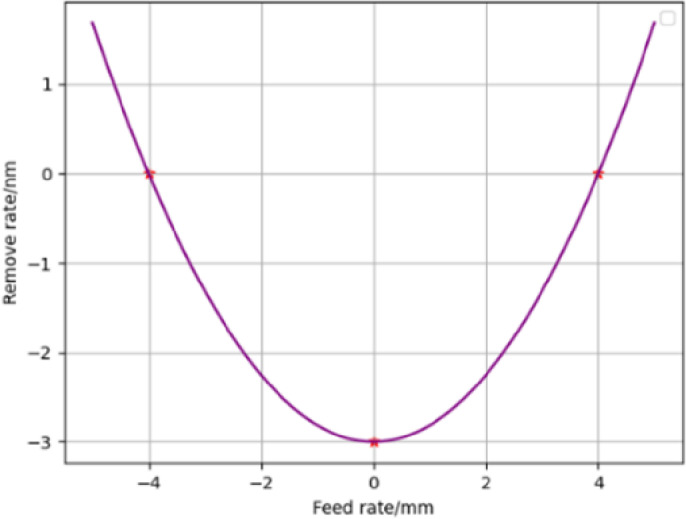
Simulated removal contour.

According to the actual situation of processing, in the stage of mirror finish polishing, take the workpiece speed 3 r/min, the grinding head speed 1800r/min, and the grinding head feed speed is 10mm/min. Use the formula (14) to simulate the removal function of the grinding head under the above conditions, as shown in Fig 3.

The removal profile obtained by modeling is a perfect approximate Gaussian distribution function as seen in Fig 3, with the location of the maximum removal depth always located at the center.

The residence time is solved with the removal as a two-dimensional convolution of the removal function and the residence time, indicated by “**”, as shown in equation (15).


$$H\left(x,y\right)=R\left(x,y\right)**D\left(x,y\right)$$ (15)

**Fig 4:**
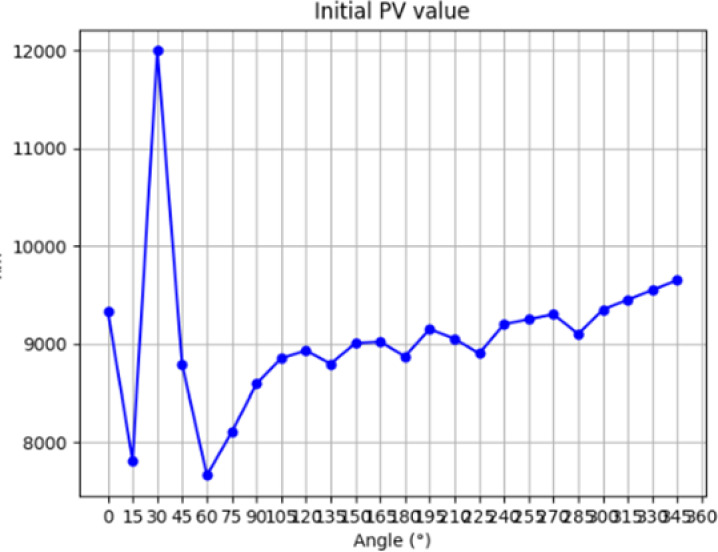
Initial PV value.

**Fig 5:**
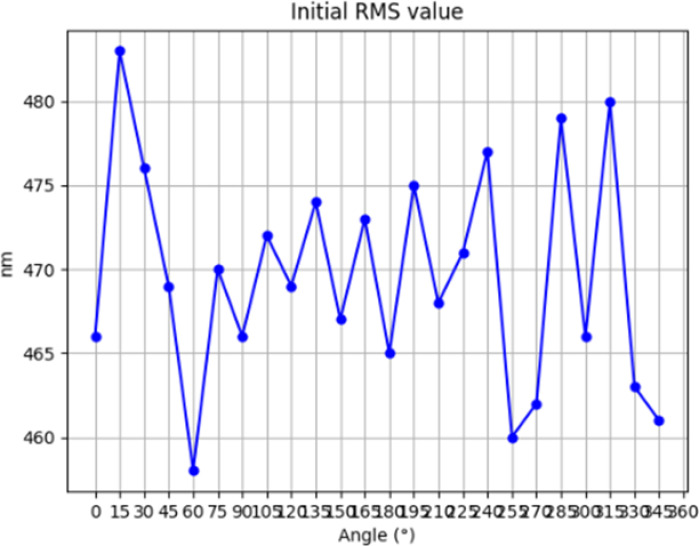
Initial RMS value.

In the polishing process, H (x, y) is the amount of material removed from the polished workpiece, which is a known quantity and can be determined by the detection device, R (x, y) is the removal function of the polished workpiece, which has been derived from the above theory, the resulting dwell time D (x, y) can be derived from the appropriate algorithms, and the algorithms commonly used in the CCOS technology at present include the Fourier Transform Method, Convolutional Iteration Method, and Matrix Algebraic Method, etc. The present experiment utilizes the TSVD-based regularization method to solve for the dwell time, and then conducts processing simulation experiments to verify the correctness of the solution. In this experiment, the TSVD-based regularization method is used to solve the dwell time, and the dwell time solved is subjected to machining simulation to verify its correctness. The initial surface error of the workpiece is measured, and the distribution of its initial surface error is shown in Figs 4 and 5, with PV = 8948nm and RMS = 470nm.

**Fig 6:**
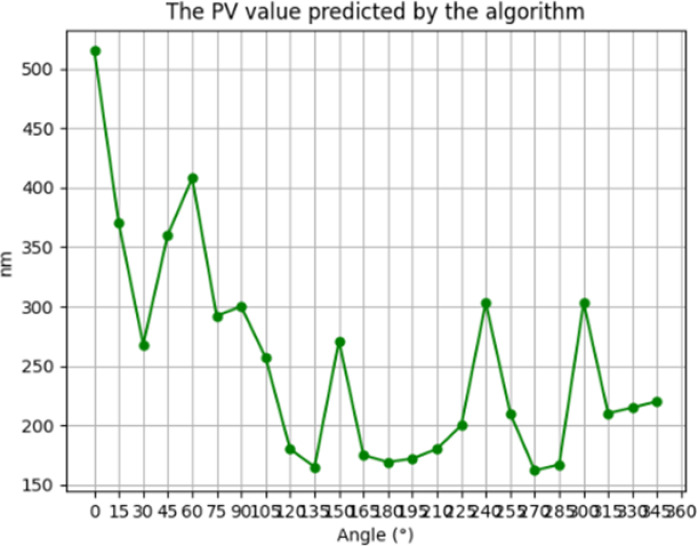
The residual of the TSVD regularization method simulates the PV value.

**Fig 7:**
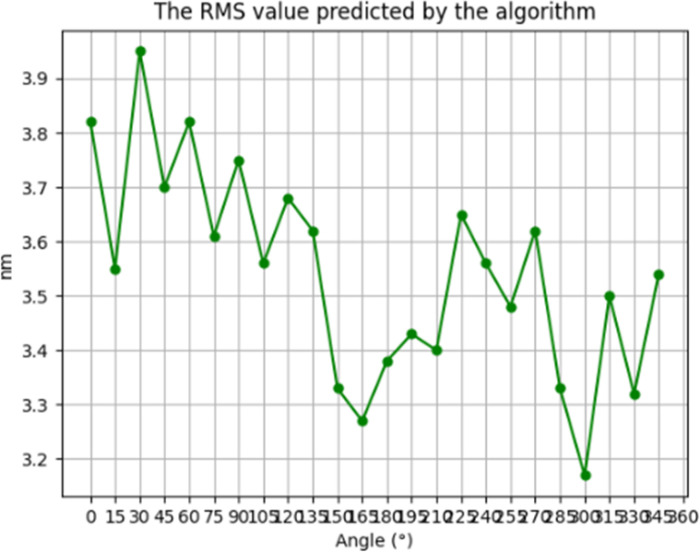
The residual of the TSVD regularization method simulates the PV value.

Figs 6 and 7 shows the simulation results of the residual error distribution obtained using the residence time algorithm based on the TSVD regularization method PV = 253nm and RMS = 3.5nm.

The formula for the convergence ratio of the face shape error is:


$$\eta =(PV{\left(RMS\right)}_{before}-PV{\left(RMS\right)}_{after})/PV{\left(RMS\right)}_{before}$$ (16)

Then, according to Eq. (16), the TSVD regularization method based on the stationary the convergence ratios of the time algorithm face shape error PV and RMS values are respectively 99% and 97%, both of which satisfy the actual processing requirements for convergence ratios.

## 5. Optical swept-incidence mirror processing radial adjustment fastening device

**Fig 8:**
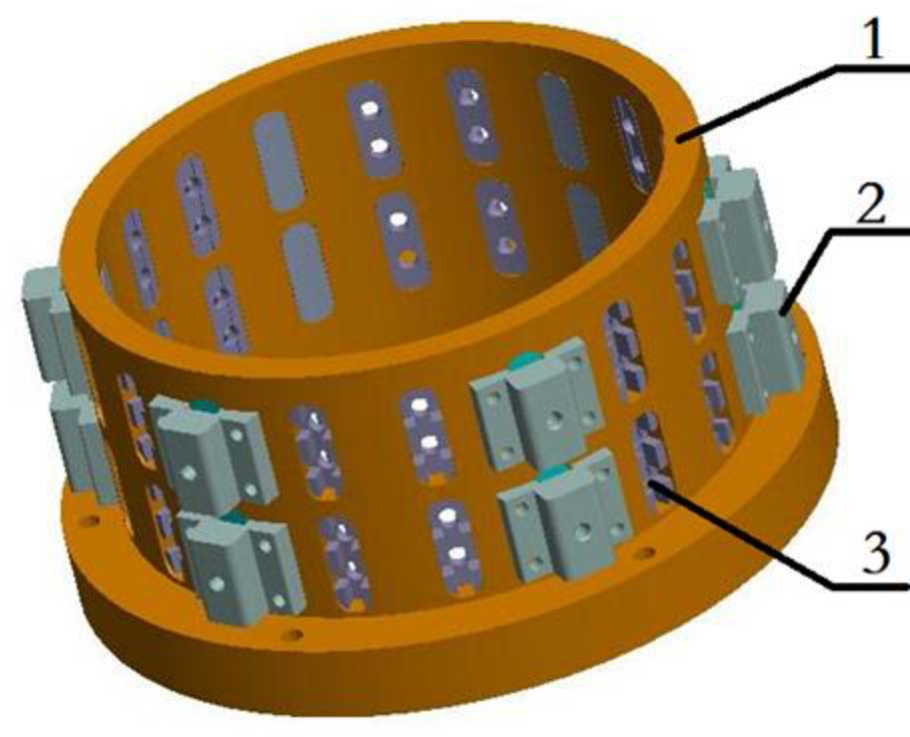
Radial adjustment fixing device.

High-resolution optical system requires ultra-high precision, difficult in processing and mounting, in the process of processing optical mirrors, optical mirrors and fastening seat aluminum alloy cavity wall need to have clearance between the fit, the fit gap can not be too small, the gap is too small will produce the workpiece deformation, bursting, cracking, nibbling knife and other unfavorable to the phenomenon of ultra-precision machining, a single side of the distance of about 0.2mm, the diameter of about 0.4mm.Optical mirrors in the Stress release after heat, the use of optical adhesive melt state fastening, in the middle of the processing break, the mirror will sink at a rate of 0.25μm/h, affecting the mirror precision. In order to solve the problem of eccentricity, an optical swept-incidence mirror machining radial adjustment fastening device was designed, as shown in Fig 8.

1-fastening seat 2-radial adjustment structure.

3-solid rubber block

### 5.1. Structural components

Ultra-precision optical swept-incidence mirror processing radial adjustment fastening device consists of three parts: fastening seat, radial adjustment structure and glue block. Fastening seat on the uniform distribution of two rows of holes, each row of 18, of which each row of 6 radial adjustment holes for radial adjustment and secondary injection fastening, 60^°^ uniform distribution, 12 holes for the first injection of glue fastening, each radial adjustment holes on both sides of the two radial adjustment structure connection holes for radial adjustment structure and fastening seat solid connection; radial adjustment structure includes radial adjustment block, adjusting screws, aluminum alloy pressure block, PTFE pressure block, aluminum alloy pressure block and PTFE pressure block glued to form a pressure block group, PTFE pressure block and mirror convex contact, radial adjustment block with 4 screws fixed in the fastening seat, adjusting screw in the center of the radial adjustment block threaded holes, fine-tuning pressure block group through the spiral displacement, fine-tuning of the mirror in the radial position of the fastening device; optical adhesive through the two injection holes into the injection groove, so that the end of the solid adhesive block and mirror convex glue together The optical glue enters the injection slot through 2 injection holes, so that the end surface of the solid glue block is glued together with the convex surface of the mirror, and the glue in the glue storage slot is glued together with the side wall of the hole of the fastening block through the side wall groove of the solid glue block to fix the radial and axial position of the mirror in the radial adjustment of the fastening device in the processing of ultra-precision optical swept-incidence mirrors.

### 5.2. Principles of operation

The left end of the fastening seat is connected to the machine chuck through the bolt group, the mirror to be processed is loaded into the fastening seat, the radial adjustment structure is loaded into the fastening seat, the gap of the mirror in the fastening seat and the coaxiality of the mirror's axis and the axis of the fastening seat are adjusted through the adjusting screws and controlled to be within 0.1 μm, and the solid glue blocks are loaded into the holes of the fastening seat sequentially to carry out the primary injection of the solid glue for fixing and tightening, and after the completion of the primary injection of the solid glue, all the radial adjusting structures are removed, and the position of the radial adjusting structure is fixed during processing. After the completion of the first injection, remove all the radial adjustment structure, sequentially in the radial adjustment holes into the solid glue block, for the second injection of glue, fix the mirror in the ultra-precision optics swept-incidence mirror processing radial adjustment of the position of the fastening device, to achieve the radial positioning of mirrors in the processing of the radial positioning and axial positioning.

## 6. Optical grazing incidence mirror processing and inspection

### 6.1. Optical grazing mirror processing

**Fig 9:**
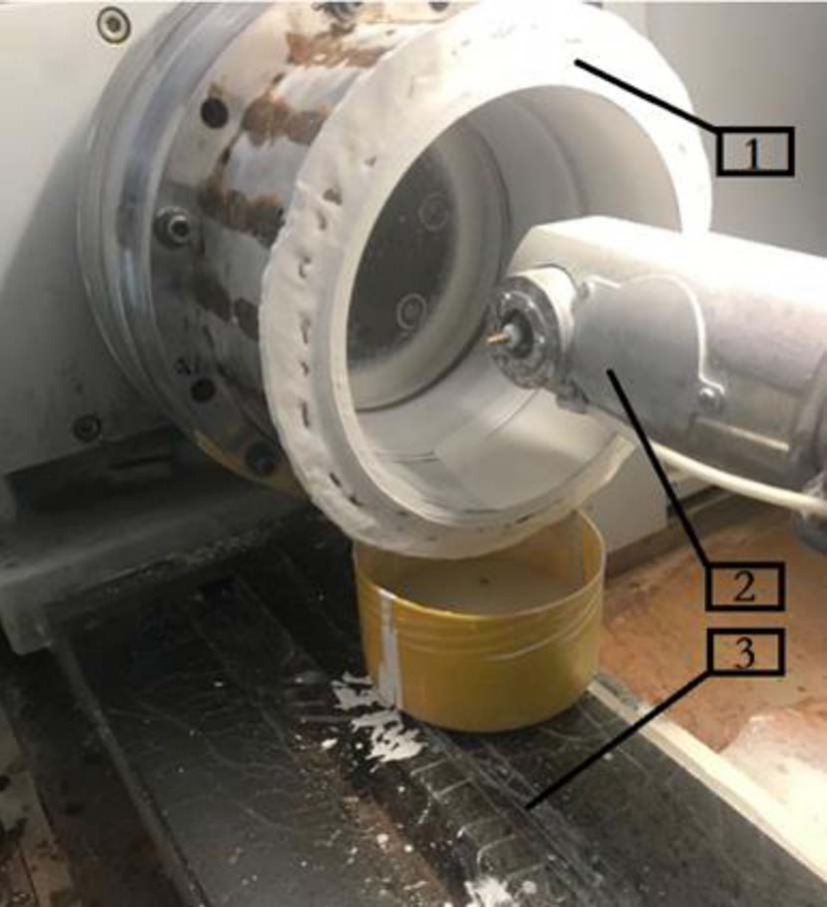
Processing equipment.

Machining is carried out on a precision universal grinding machine kel-vista ur175/600 using a self-developed radially adjustable fastening device, automatic supply of polishing fluid device. The processing equipment is shown in Fig 9. Abrasive water flow polishing requires a long processing time, should ensure that the water jet polishing system to meet the abrasive water flow polishing fluid to be stable for a long time. Through the polishing fluid on-line detection and control of recycling, filtration and reuse, etc., to control the concentration of polishing fluid, viscosity, temperature, PH value and other performance indicators for a long time stable.

1-radial adjustment fastening device 2-automatic polishing liquid supply device 3-universal grinder.

Workpiece rotational speed 3-5r/min, grinding head rotational speed 1800r/min, grinding head unidirectional feeding, feeding speed 4-20mm/min. The end of the grinding head is a polyurethane cylinder of φ20mm, 20mm thick, which is in close contact with the mirror surface during machining, and the pressure is 0.5 N. Three rounds of polishing are carried out, and the test is carried out to adjust the parameter of the removing function after each round of polishing. The first round of 160h rough polishing with cerium oxide of W2, removing peaks larger than 1500nm, the second round of 720h fine polishing with cerium oxide of W1, removing peaks larger than 800nm, the third round of 720h super fine polishing with cerium oxide of W0.8, removing peaks larger than 200nm.

### 6.2. Optical grazing incidence mirror surface shape error detection

Optical grazing incidence mirrors were inspected on a taylor hobson profiler Form Talysurf PGI. Each busbar was sampled at an interval of 0.5 mm, and one busbar was scanned at 15^°^ intervals, for a total of 24 busbars, which were measured using the 24-bar method.

**Fig 10:**
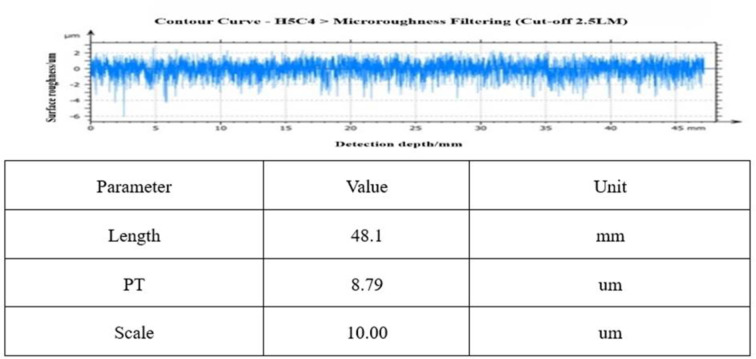
Mirror accuracy before rough casting.

Fig 10 shows the roughness value of one of the busbar profiles before rough polishing, from which it can be seen that the roughness value fluctuates within ± 4.5 μm, which is a very rough surface for an optical mirror. Measured average values: RMS: 470nm, PV: 8948nm, Rq: 581nm.

**Fig 11:**
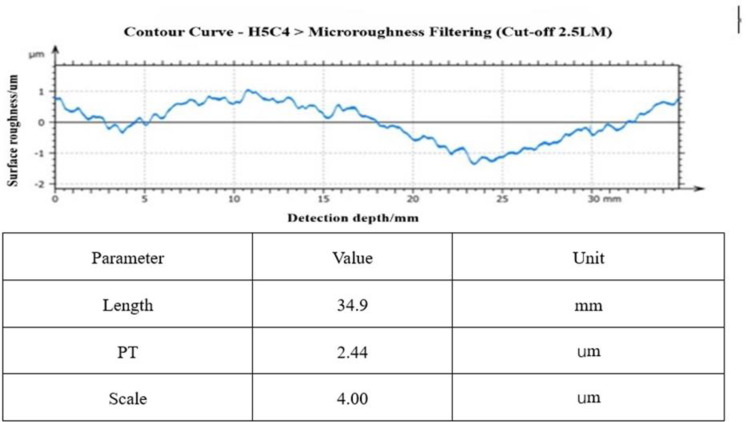
160h coarse polishing mirror precision.

After the first round of 160h rough polishing with cerium oxide with W2, Fig 11 shows the surface roughness value of one of the busbars of the mirror surface after rough polishing, from which it can be seen that the roughness value fluctuates within ± 1.5 μm, and the surface error after rough polishing has been effectively converged, and the average values measured: RMS:23.8 nm, PV:2566 nm, Rq:30.2 nm.

**Fig 12:**
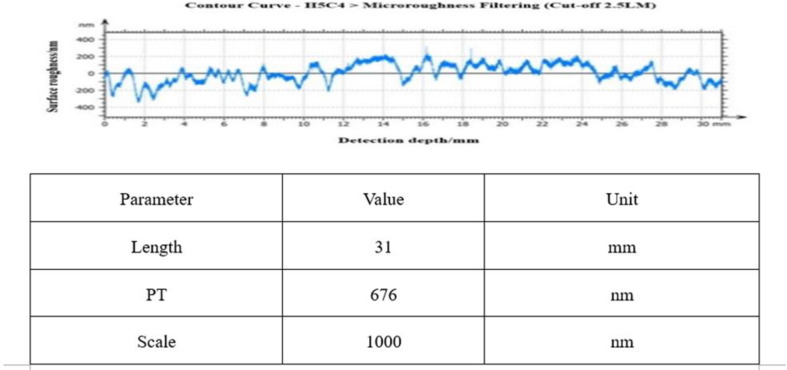
720h fine polishing mirror precision.

The second round of 720h fine polishing with cerium oxide of W1, Fig 12 shows the surface roughness value of one of the busbars of the mirror surface after fine polishing, as can be seen from the figure, the roughness value fluctuates within ± 400nm, and the effect of the surface error is improved obviously after the fine polishing, and the measured average values: RMS:12nm, PV:1056nm, Rq:14.9nm.

**Fig 13:**
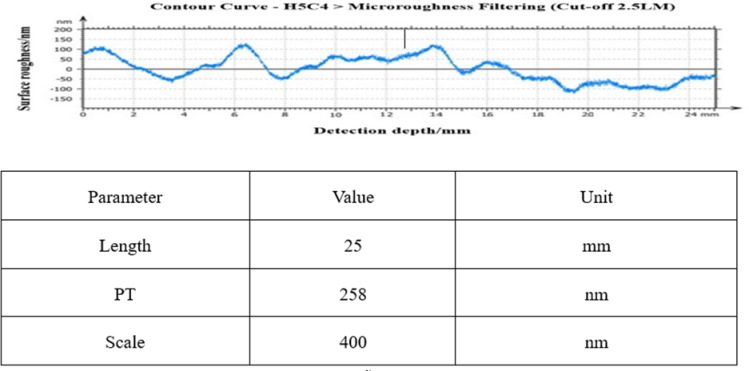
720h ultra fine polishing mirror precision.

The third round of cerium oxide with W0.8 was used for 720h super-finishing polishing. Fig 13 shows the surface roughness value of one of the busbars after superfine polishing, from which it can be seen that the roughness value fluctuates within ± 100 nm, which has met the requirements of the composite extreme ultraviolet-soft X-ray telescope, and the measured average value: RMS: 3.5 nm, PV: 253 nm, Rq: 4.6 nm.

**Fig 14:**
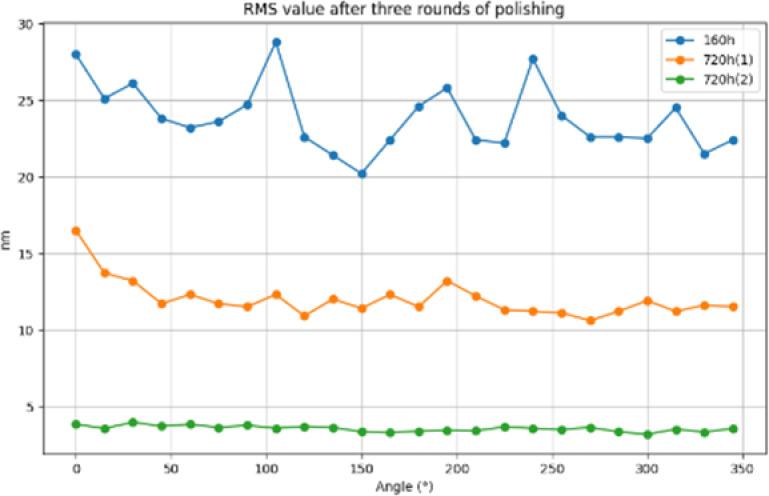
RMS value comparison.

**Fig 15:**
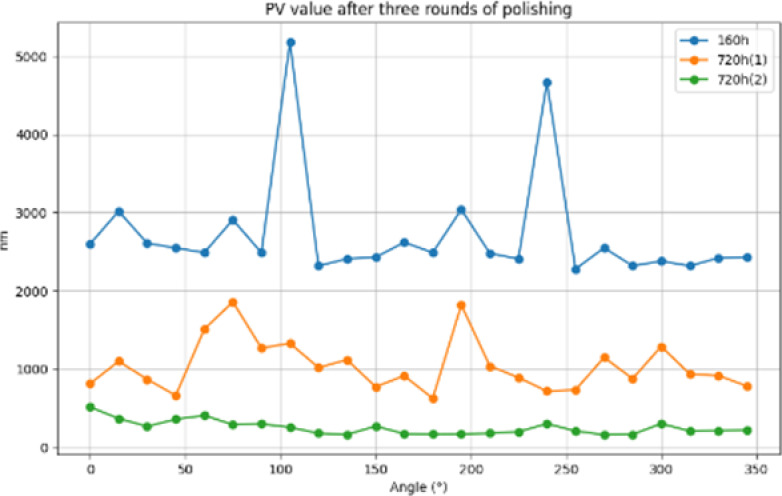
PV value comparison.

**Fig 16:**
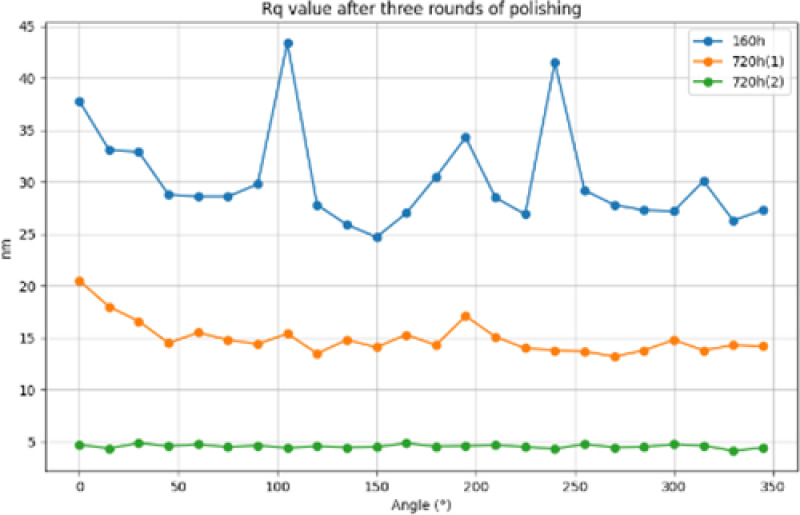
Rq value comparison.

After three polishing, 24 busbar detection comparison chart, RMS value comparison shown in Fig 14, PV value comparison shown in Fig 15, Rq value comparison shown in Fig 16, from the three indicators of change, it can be clearly seen that the face shape accuracy and surface roughness have been effectively converged, and the final machining result meets the requirements to prove the feasibility of the processing method.

## 7. Conclusion

In the design of the solar soft X-ray-extreme ultraviolet band composite telescope, the machining of the inner surface of the coaxial confocal Wolter-I is one of the core technologies, and the effects of surface roughness and face shape error on imaging are analyzed, and the radial adjusting fastening device and the device of automatic supplying polishing liquid are designed. Through the experiments, 160h rough polishing, 720h fine polishing and 720h super fine polishing were carried out with grain sizes W2, W1 and W0.8 respectively, and the mirror precision PV, RMS and roundness root-mean-square error measurements were carried out through non-contact by utilizing the 24-bus method, and the final results of the measurements were RMS: 3.5 nm, fluctuating 0.78 nm; PV: 253 nm, fluctuating 291 nm; Rq: 4.6nm, fluctuating 0.73nm. The results show that the proposed processing method is completely feasible in the ultra-precision processing of swept-incidence mirrors, which provides a guarantee for further improving the performance of swept-incidence mirrors.
